# Strangulated sliding spigelian hernia: A case report

**DOI:** 10.1016/j.ijscr.2018.10.043

**Published:** 2018-11-16

**Authors:** P.O. Igwe, N.A. Ibrahim

**Affiliations:** a Department of Surgery, University of Port Harcourt Teaching Hospital(UPTH), Alakahia, Port Harcourt, Rivers State, Nigeria; b Department of Surgery, Lagos State University Teaching Hospital (LASUTH), Ikeja, Lagos State, Nigeria

**Keywords:** Strangulated sliding spigelian hernia, Nylon darn

## Abstract

**Introduction:**

Spigelian hernia is uncommon and frequently presents with features of intestinal obstruction. Clinical diagnosis is difficult in patients without obvious abdominal mass and a computed tomography scan is very helpful in making diagnosis. We report a patient who presented with a strangulated sliding left sided spigelian hernia who was pre-operatively diagnosed and successfully managed surgically.

**Presentation of case:**

A 56-year-old woman was to referred to the surgical emergency unit of our institution with abdominal swelling and pain of three days. There was no abdominal distension or fever and had no history of recurrent abdominal pains, abdominal swelling or surgery. Examination revealed a middle aged woman with a tender mass measuring about 10 cm by 8 cm in the left iliac fossa region of the abdomen. Bowel sounds were hyperactive and digital rectal examination revealed an empty rectum consistent with the diagnosis of acute intestinal obstructions. Investigations done by the patient at the referring hospital included a computed tomography (CT) scan which was suggestive of an obstructed Spigelian hernia with the sac containing a small bowel loop and mesentery.

She had emergency herniorrhaphy and findings were herniation through a facial defect of about 5 cm by 4 cm along the lateral border of the rectus sheath with the hernia sac containing sero-sanguineous peritoneal fluid, gangrenous ileal segment and part of herniated urinary bladder forming the lower wall of the sac. Resection of gangrenous bowel with an end to end anastomosis was carried out, viable contents of the sac were reduced and defect repaired with interrupted non absorbable sutures to approximate the internal oblique and transversus abdominis to rectus sheath. In addition, nylon darning from inguinal ligament to the rectus sheath was also done. Her post-operative recovery was satisfactory and she remained well six months after surgery.

**Conclusion:**

Pre-operative diagnosis of strangulated Spigelian hernia is difficult but can be aided by a CT. The use of nylon darning to re-inforce the repair can be rewarding where mesh cannot be used or unavailable.

## Highlights

• The finding of both sliding and strangulating spigelian hernia is rare.
• Nylon darning was used for the repair rather than mesh because it was a clean contaminated surgery.
• The approximation was the conjoint tendon to rectus sheath after primary closure of defect.

## 1 Introduction

Spigelian hernias are named after Adriaen Van den Spigehel, an anatomist from Belgium who described the fascial defects associated with this condition [1]. They are also called spontaneous lateral ventral hernias, hernia of the semilunar line, or hernias through the conjoint tendon. The incidence is approximately 2% of abdominal wall hernias with a slightly higher occurrence in the female sex and can be congenital or acquired [2].

Spigelian hernias are thought to result from fascial weakness related to perforating vessels. Some authorities suggested that up to 50% of these hernias result from previous abdominal operations that weaken the semilunar line prompting herniation [3]. Factors that may lead to increased tension on the abdominal wall aponeurosis or increase intra-abdominal pressure, such as straining due to bladder outlet obstruction, chronic cough, obesity or multiple pregnancies are also believed to predispose patients to the development of Spigelian hernia. A viscous, lipoma or omentum may be a leading point which gradually results in herniation [4].

A high index of suspicion is required to make diagnosis of this rare entity. Abdominal ultrasonography is useful while Computerized Tomography (CT) scan of the abdomen with contrast has become the best imaging method in confirming the diagnosis especially when in doubt [5].

Spigelian hernia requires surgical repair to prevent strangulation while those presenting with complications need immediate surgery. We report the first case of strangulated sliding spigelian hernia in a middle-aged woman seen in our institution. This work has been reported in line with the SCARE criteria [6].

## 2 Presentation of case

A 56-year-old woman was referred to the surgical emergency of our institution with abdominal swelling and pain of three days duration. She vomited recently ingested food twice prior to presentation. There was no abdominal distension or fever. She had no history of recurrent abdominal pains, abdominal swelling or surgery.

**Fig. 1:**
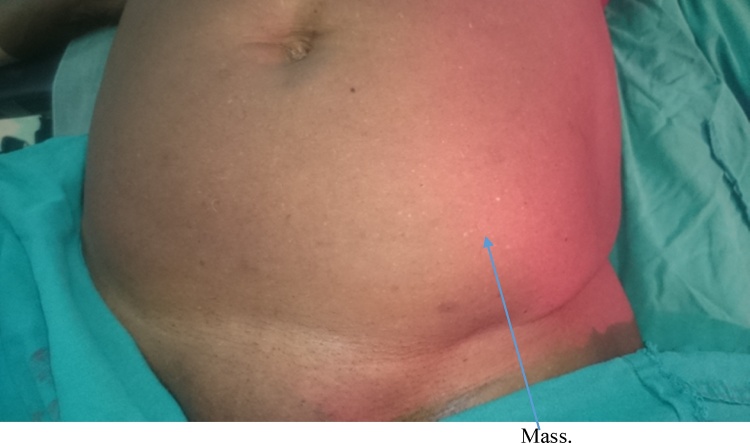
Mass in the lower left abdomen.

**Fig. 2:**
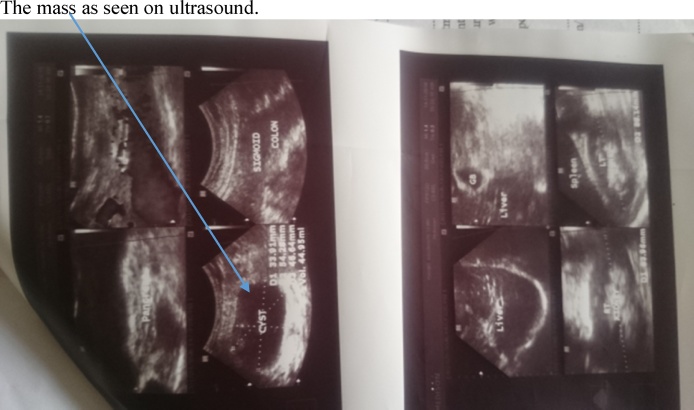
Ultrasound showing the mass appearing cystic.

**Fig. 3:**
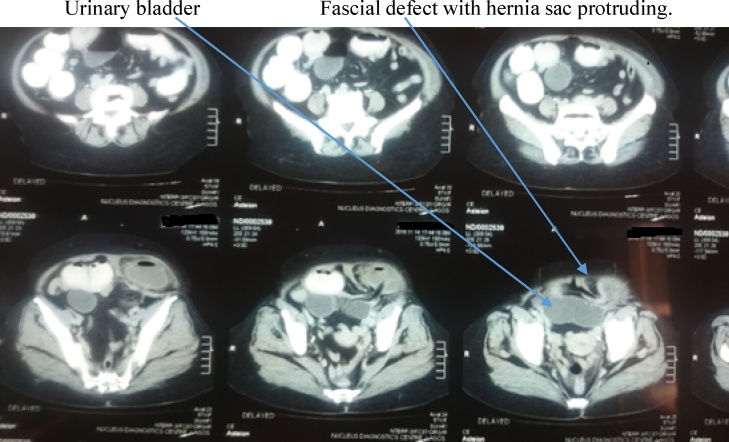
Computerized axial tomographic scan showing the hernia.

Examination revealed a middle aged woman in no painful or respiratory distress. She was afebrile, anicteric and not pale or dehydrated. Her pulse rate was 80 beats per minute regular and of good volume. Blood pressure was normal while temperature was 37 °Celsius. A tender mass measuring about 10 cm by 8 cm was present in the left iliac fossa region of the abdomen (Fig. 1). Bowel sounds were hyperactive and digital rectal examination revealed an empty rectum consistent with the diagnosis of acute intestinal obstructions. Investigations done by the patient at the referring hospital included an abdomino-pelvic Ultrasound scan which revealed a cystic mass of about 45mls volume suggestive of mesenteric hematoma (Fig. 2) and a computed tomography (CT) scan which was suggestive of an obstructed spigelian hernia with the sac containing a small bowel loop and mesentery (Fig. 3). Other investigations carried out included estimation of serum Haemoglobin, electrolytes, urea and creatinine levels which were all within normal range. She also had chest radiograph which was normal.

**Fig. 4:**
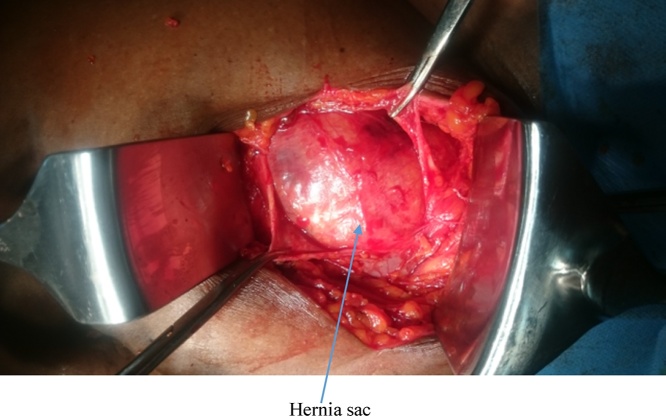
Spigelian hernal sac.

**Fig. 5:**
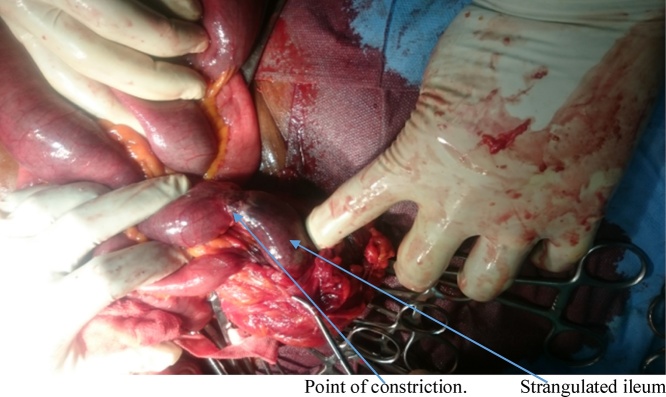
Strangulated segment of ileum.

**Fig. 6:**
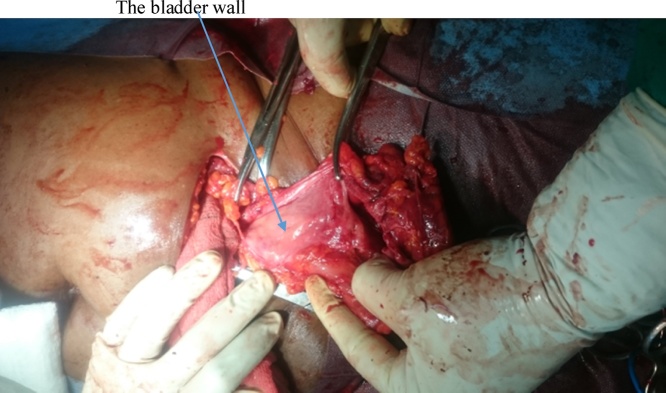
Viable bladder forming part of the wall of sac.

**Fig. 7:**
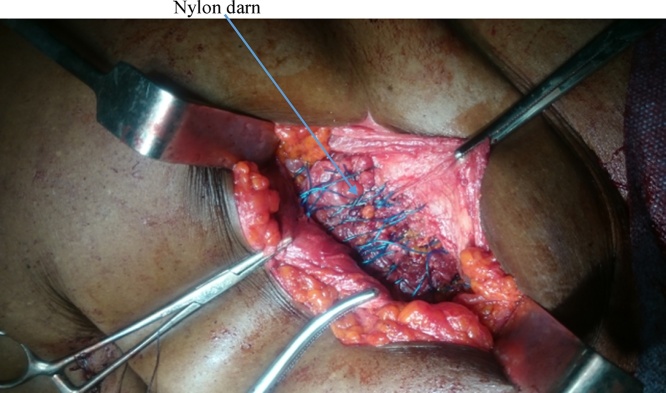
Nylon darn.

**Fig. 8:**
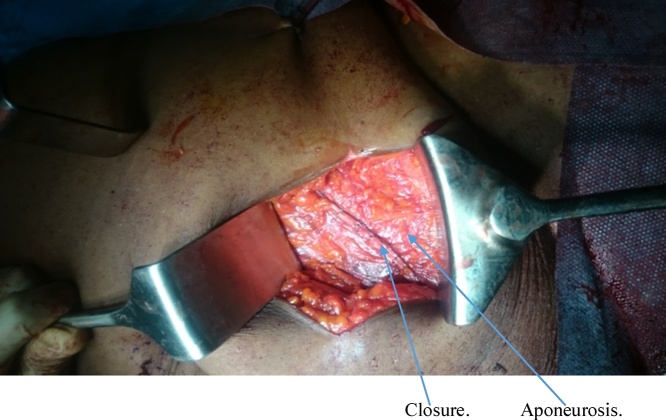
Closure of the external oblique aponeurosis.

She had emergency herniorrhaphy within two hours of admission after satisfactory resuscitation. Under general anaesthesia, a transverse left lower abdominal incision was made and findings were herniation through a facial defect of about 5 cm by 4 cm along the lateral border of the rectus sheath (Fig. 4). Hernia sac contained sero-sanguineous peritoneal fluid, gangrenous ileal segment (Fig. 5) and part of herniated urinary bladder forming the lower wall of the sac (Fig. 6). Resection of gangrenous bowel with an end to end anastomosis was carried out. Other viable contents of the sac were reduced and defect repaired with interrupted non absorbable sutures to approximate the internal oblique and transversus abdominis to rectus sheath. In addition, nylon darning from inguinal ligament to the rectus sheath was also done (Fig. 7). Then closure of external oblique aponeurosis. (Fig. 8). Her post-operative recovery was satisfactory and she remained well six months after surgery.

## 3 Discussion

Spigelian hernia is a rare type of ventral abdominal wall hernia which occur secondary to a defect in the transversus abdominis muscle and rectus sheath aponeurosis allowing abdominal contents to herniate through the linea semilunaris. It often starts as a protrusion of pre-peritoneal fat through the hernia ring, a well-defined defect in the Spigelian aponeurosis at the “Spigelian hernia” belt. This is the widest part of the aponeurosis where 85–90% of the hernias occur and lies between 0 and 6 cm cephalad to the inter-spinous plane [4].

Herniation is very rare above the umbilicus because the semilunar line is supported by the aponeurosis of the external oblique on the anterior aspect and posteriorly in the cranial two thirds by the transversus abdominis muscle which is muscular almost to the midline in the upper abdomen. Hernia sac is found in most cases and contents are commonly greater omentum, small intestine as in present case or part of the colon. Rare contents of the sac include acutely inflamed appendix, Crohn’s appendicitis and an incarcerated Meckel’s Diverticulum. Bilateral Spigelian hernias and Richter type of Spigelian hernia have also been reported [4,5]. Such type of hernias have also being reported following laparoscopic procedure, through a pre-existing fascial weakness, that became obvious as a result of the pneumoperitonem [7]. The sliding form where part of the sac is formed by an intra-abdominal viscera such as the urinary bladder as seen in the present case, has not been reported in the English literature.

Peak incidence of spigelian hernia is between the 4th and 7th decade of life and occurs mostly on the right side. The presenting symptoms are commonly abdominal pain, an intermittent swelling in the anterior abdominal wall or signs of intestinal obstruction [8]. It is reported that about 21% may present with small bowel incarceration [9]. Pre-operative clinical diagnosis is possible in patients with palpable mass along the Spigelian aponeurosis, however, this may be difficult in those presenting with non-specific abdominal pains and have no visible or palpable mass due to reduction of hernia sac content or presence of intramural or inter parietal hernia [4, [9]. This condition may mimic other lesions in the abdominal wall such as rectus sheath hematoma, seroma, parietal abscess, lipoma, peritoneal tumour implants and pseudocyst at the end of the ventriculoperitoneal shunts [4]. It is reported that only 50% of cases are diagnosed pre-operatively [10].

Plain abdominal X-rays are not specific and can only show features of bowel obstruction in those presenting with intestinal obstruction. Ultrasound scanning is recommended as first line imaging investigation in suspected cases. It is rapid, fairly accurate, non-invasive and relatively easy to perform. It has a sensitivity and positive predictive value (PPV) of 90% and 100% respectively. However, drawbacks include being operator dependence and reduced diagnostic accuracy in obese patients. It is advocated that scanning of the semilunar line should be done in all patients with obscure abdominal pain and swelling of the abdominal wall in both supine and erect positions and while patient performs a Valsalva maneuver to increase accuracy [4,10,11]. Presently, abdomen and pelvis CT scanning with contrast is the best and most reliable method to make the diagnosis in doubtful cases [11]. It has sensitivity and PPV of 100% respectively and also provides additional information about different layers of abdominal wall and surrounding soft tissue changes. In addition, bowel strangulation can be better identified on a CT [11]. This imaging modality may not be readily available in resource-poor countries due to cost thereby limiting the use in diagnosis. Magnetic Resonance Imaging (MRI) is becoming more available and may be of help in the preoperative diagnosis of difficult cases.

Prompt surgical repair is recommended for spigelian hernia to prevent subsequent strangulation while those presenting with bowel obstruction will require emergency surgery. Open or laparoscopic approach could be employed depending on experience and availability of laparoscopy facilities. Techniques of open repair followed the trend in repair of hernias generally with simple closure of the defect in the form of hernioraphy in earlier reports [1]. Hernioplasty, by use of synthetic mesh or fascia lata graft to achieve tension free repair, was later adopted for the repair of spigelian hernia [13,14]. It is particularly useful for repair of hernias with large defect. Synthetic mesh repair is, however, not recommended in emergency situations with contaminated field following strangulation. We avoided use of mesh and did tension free nylon darn repair in addition to initial simple closure of the defect in this case we are reporting.

Laparoscopic approach has been widely utilised in both elective and emergency repair of the hernia using intra-peritoneal onlay mesh (IPOM), trans-abdominal pre-peritoneal (TAPP) and total extra-peritoneal (TEP) techniques [10]. It offers faster recovery and lower morbidity [5]. The first intra-abdominal laparoscopic repair of spigelian hernia was performed by Carter and Mizes in 1992 using sutures to close the defect [12]. Preperitoneal laparoscopic repair is suitable for Spligenian hernia because the defect in the aponeurosis is better identified in the preperitoneal plane. The use of laparoscopy is currently gaining ground in developing economy. This method may be adopted in subsequent repair especially in uncomplicated cases.

## 4 Conclusion

Sliding Spigelian hernias are not common and clinical diagnosis requires a high index of suspicion. Strangulation is common and occurs in about a quarter. Imaging studies, especially CT may aid pre-operative diagnosis. Surgery is the modality of treatment and open approach is often used where facilities and expertise for laparoscopy are not available. Tension free repair with mesh is recommended. However, in cases with strangulation where use of mesh may not be advisable or where mesh is not readily available, nylon darning may be an acceptable alternative.

## Conflicts of interest

No Conflict of interest.

## Sources of funding

No Source of Funding.

## Ethical approval

Exemption of Ethical approval was given because no identifiable patient’s parts were seen.

## Consent

Written and signed consent to publish as case report was obtained from patient.

## Author contribution

PO Igwe; Case Design and write up.

NA Ibrahim; Surprvised, proof read and approve with corrections.

## Registration of research studies

Case report.

## Guarantor

PO Igwe.

## Provenance and peer review

Not commissioned, externally peer reviewed.
